# Chemotherapy Controls Metastasis Through Stimulatory Effects on GRP78 and Its Transcription Factor CREB3L1

**DOI:** 10.3389/fonc.2020.01500

**Published:** 2020-09-11

**Authors:** Annat Raiter, Julia Lipovetsky, Lucila Hyman, Shany Mugami, Tali Ben-Zur, Rinat Yerushalmi

**Affiliations:** ^1^Felsenstein Medical Research Center, Petach Tikva, Israel; ^2^Sackler Faculty of Medicine, Tel Aviv University, Tel Aviv, Israel; ^3^Department of Pathology, Rabin Medical Center, Beilinson Hospital, Petach Tikva, Israel; ^4^Davidoff Cancer Center, Rabin Medical Center, Petach Tikva, Israel

**Keywords:** glucose-regulated protein 78, CREB3L1, triple-negative breast cancer, chemotherapy, metastasis

## Abstract

To achieve a cure for metastatic breast cancer, further understanding of molecular drivers of the metastatic cascade is essential. Currently, chemotherapy regimens include doxorubicin and paclitaxel which act in part by inducing the unfolded protein response (UPR). The master regulator of the UPR, glucose regulated protein 78 (GRP78), localizes on the surface of tumor cells and is associated with metastatic disease. Cyclic AMP responsive element binding protein 3-like 1 (CREB3L1), a member of the UPR, is a breast cancer metastasis suppressor that acts on cyclic AMP to promote the expression of target genes including GRP78. The aim of the present study was to evaluate the effects of chemotherapy on CREB3L1 and cell-surface GRP78 expression and its association with the development of breast cancer metastasis. For this purpose, we use breast cancer cells migration *in vitro* assays and an *in vivo* metastatic mouse model. The results showed that chemotherapy activated CREB3L1 and enhanced cell-surface GRP78 expression specifically in triple-negative breast cancer cells (TNBC), reducing their migration and metastatic potential. CREB3L1 knockout (KO) in the triple negative MDAMB231 cell line using CRISPR/Cas9 technology led to inhibition of GRP78 expression and abrogation of the CREB3L1 metastatic suppression function. Inoculation of CREB3L1-KO MDAMB231 cells into a mouse metastatic model induced a massive metastatic profile which chemotherapy failed to prevent. These findings elucidate a potential pathway to the development of a novel treatment strategy for metastatic TNBC based on modulating CREB3L1 and cell-surface GRP78 expression by chemotherapy and GRP78-targeted drugs.

## Introduction

The 5-year survival rate of metastatic breast cancer is currently only 27% (1, 2) despite considerable progress in all treatment modalities (3–5). To achieve complete cure, comprehensive investigation of the molecular drivers that promote tumor shrinkage in metastatic disease is essential.

Chemotherapy remains the main treatment modality for metastatic triple-negative breast cancer (TNBC) (6). Anthracyclines and taxanes have been proven effective through decades of clinical practice. Doxorubicin and paclitaxel are known to act in part by inducing the unfolding protein response (UPR) in cancer cells through the endoplasmic reticulum stress pathway (7–9). The UPR increases the folding capacity of the endoplasmic reticulum, reduces translation of new proteins, and enhances the degradation of misfolded proteins. If UPR functions fail to complete, stress is prolonged, resulting in signaling for cell apoptosis (10–12). Glucose-regulated protein 78 (GRP78), a master regulator of the endoplasmic reticulum stress response, has been further characterized as a chaperone in the cytoplasm of cancer cells as well as a signaling receptor in the cell plasma membrane (13–17). The different cellular localization patterns of GRP78 are associated with different signaling pathways that regulate apoptosis, cell invasion, and metastatic development (18, 19). GRP78 overexpression is associated with the progression of metastatic epithelial tumors through epithelial-to-mesenchymal transition signaling (20). Earlier, our group demonstrated that doxorubicin treatment induces the translocation of GRP78 from the cytoplasm to the surface of breast cancer cells, which facilitates their tagging for apoptosis (21).

Studies have shown that cell-surface GRP78 expression correlates with the expression of cyclic AMP responsive element binding protein 3-like 1 (CREB3L1) which has been identified as a suppressor of metastasis in breast cancer (22, 23). CREB3L1 is the most recently identified member of the UPR signaling pathway and contains both a basic leucine zipper (bZIP) and DNA-binding domain (24). Furthermore, it has been characterized as an endoplasmic reticulum transmembrane protein activated in a similar manner to the activating transcription factor 6 (ATF6) (25–27). The mature activated form is a transcription factor that acts on both endoplasmic reticulum stress responsive elements and cyclic AMP responsive elements to increase the expression of target genes, including GRP78 (24). Recent reports suggest that doxorubicin inhibits tumor cell proliferation through proteolytic activation of CREB3L1 and not by induction of DNA damage (28). Paclitaxel has been reported to increase the production of ceramide which promotes the trafficking of CREB3L1 from the endoplasmic reticulum to the Golgi complex (29).

The aim of the present study was to evaluate the effects of chemotherapy on CREB3L1 and cell-surface GRP78 expression and significance in a metastatic setting. We hypothesized that chemotherapy drugs trigger endoplasmic reticulum stress and promote cell-surface expression of GRP78 and CREB3L1 activation through the UPR pathway, thereby preventing progression of tumor metastasis.

## Materials and Methods

### Human Breast Cancer Cell Lines and Culture Conditions

Four human breast cancer cell lines were used: MDAMB468 and MDAMB231 are HER2-neu-, estrogen receptor (ER)-, and progesterone receptor (PR)-negative; BT474 is ER-, PR-, and HER2-neu positive; and HM7 is ER-positive and HER2-neu-negative. Cells were grown in DMEM supplemented with 10% FCS, l-glutamine (2 mM), Na-pyruvate (1 nM), penicillin (100 μg/ml), streptomycin sulfate (0.1 mg/ml), and nystatin (12.5 μg/ml) (Complete Culture Medium, Biological Industries, Kibbutz Beit HaEmek, Israel). Cultures were maintained at 37°C in a humidified 5% CO_2_ incubator. A large stock of cells was prepared to maintain the homogeneity and tumorigenicity of the cell lines.

### Cell Line Authentication

CREB3L1 knockout (KO) MDAMB231 cells were generated using CRISPR/Cas9 and maintained by puromycin selection. CREB3L1-KO cells were monitored with fluorescence activated cell sorting (FACS) using a specific antibody to determine CREB3L1 expression. Cells were not used beyond passage five and were examined for *Mycoplasma* at least once every 6 months.

### Drugs

Doxorubicin, 0.01 μg/ml (Ebewe Pharma, Am Attersee, Austria) and paclitaxel, 0.01 μg/ml (Sanofi Aventis, Paris, France) were added to cultures for 24 or 48 h. The drug concentrations were determined in preliminary assays in which we tested the following different concentrations: 0.01, 1, and 5 μg/ml (21) for the determination of minimum cell cytotoxicity, <10% dead cell in cell cultures.

### Monolayer Gap Closure Assays

Tumor cell lines (400,000 cells/dish) were seeded in six-well plates until 100% confluence for 24 h. A 200 μl pipette tip was pressed firmly against the top of the tissue culture plate generating a vertical wound through the cell monolayer (30). The medium and cell debris were aspirated, and culture medium was added along with doxorubicin and paclitaxel. The wound was inspected, and an initial image was obtained. After 24 h and after treatment, images were obtained under a light microscope at 10 × magnification. Wound closure was determined by quantifying the scratch area using ImageJ v1.45 software.

### Transwell Migration Assay

Cell migration was measured using an 8.0 μm Thinsert cell culture insert for 24-well plates (Greiner Bio-One, Frickenhausen, Germany) as previously described (30). The breast cancer cell lines were incubated in six-well plates for 24 h and treated with doxorubicin or paclitaxel for an additional 24 h, as described above. Cells were detached and seeded in inserts at a concentration of 100,000 cells in 500 μl medium supplemented with 5% serum. After overnight incubation, the inserts were removed, the migrated cells were fixed with 4% paraformaldehyde and stained with DAPI (Invitrogen Molecular Probes, Thermo Fisher Scientific, Waltham, MA, USA). Photographs were taken using a fluorescent microscope at 10 × and 40 × magnification. ImageJ software was used to obtain an average cell count for each sample. Two assays were performed and each assay was repeated in triplicate.

### CREB3L1 Knockout With CRISPR/Cas9 Technology

To generate CREB3L1-KO MDAMB231 cells, we applied the Genome-Wide knockout kit using CRISPR CREB3L1 (Locus ID 90993) (Origene, Rockville, MD, USA) according to the manufacturer's instructions. The kit contains a CREB3L1 knockout coding gene and a knock-in functional cassette containing a green fluorescent protein (GFP) gene and a puromycin-resistant gene. Both target sequences are located at the 5′ end of the ORF in order to obtain precise cleavage in this region of the gene loci with gRNA vectors. Negative scrambled gRNA was used as the control. Cells were transfected using Turbofectin reagent, and GFP-positive cells resistant to puromycin (2 μg/ml) were selected at serial passages, according to the manufacturer's instructions, and maintained with the addition of puromycin. CREB3L1-KO cells were validated using FACS and western blot, as described below. The frequencies of small insertions/deletions at the on-target and putative off-target sites were measured by deep sequencing (Hylabs, Rehovot, Israel).

### FACS Analysis

To evaluate the percentage of cells expressing surface GRP78 and cytoplasmic CREB3L1, tumor cells (200,000 cells/dish) were detached using cold PBS and transferred to FACS tubes for staining. Cells were resuspended in 50 μl PBS supplemented with 5% FBS and 0.1% NaN_3_. To detect cell-surface GRP78, monoclonal anti-GRP78 antibody (DyLight® 488, Abcam, Cambridge, MA, USA) was added to tubes at a concentration of 2 μg/ml for 1 h at 4°C. To ascertain non-specific binding, isotype control was determined using IgG 2a DyLight® 488. Intracellular staining for CREB3L1 was performed initially using a FIX & PERM Cell Permeabilization kit consisting of matched Fixation and Permeabilization Reagent (R&D Systems, Minneapolis, MN, USA). Monoclonal anti-CREB3L1 antibody (1 μg/ml; Santa Cruz Biotechnology, Dallas, TX, USA) was added to the tubes for 1 h at room temperature followed by the secondary anti-mouse PE antibody (Jackson ImmunoResearch Labs, West Grove, PA, USA) for 30 min at room temperature. The isotype control used for CREB3L1-stained cells was anti-IgG1 antibody. After washing, cells were suspended in 400 μl PBS for FACS analysis. Gated stained live cells were assessed via FACS using Kaluza software (Beckman Coulter Life Sciences, Indianapolis, IN, USA).

To detect nuclear CREB3L1, tumor cells were permeabilized twice using a FIX & PERM® Cell Permeabilization kit, first for 20 min, and then, after washing, for 5 min. Cells were then washed and suspended in PBS supplemented with 5% FBS and 0.1% NaN_3_ for CREB3L1 staining, as described above. After washing, cells were suspended in 400 μl PBS for FACS analysis (30).

### Western Blot Analysis

Western blot was performed to determine CREB3L1 expression in MDAMB231 wild-type and MDAMB231 CREB3L1-KO cells, either left untreated or treated with doxorubicin and paclitaxel. Cultured cells were washed twice with PBS and lysed in radioimmunoprecipitation assay buffer for 15 min on ice. Cell lysates were clarified by centrifugation at 12,000 × g for 10 min, and protein concentrations were determined with the BCA assay (Pierce, Thermo Scientific, Rockford, IL, USA). Lysates were separated by 10% Bis-Tris gel electrophoresis, and proteins were transferred to nitrocellulose membrane and immunoblotted with an anti-CREB3L1 rabbit polyclonal antibody (Abcam, Cambridge, MA, USA). Immunoblots were visualized using infrared 800 nm dye-tagged secondary antibody (IRDye 800 CW goat anti-rabbit, LiCOR, Lincoln, Nebraska, USA); proteins were visualized using a LiCOR Odyssey infrared imager. Data were normalized against GAPDH (rabbit polyclonal antibody; Abcam) to determine the percentage of protein expression.

### Cell Immunofluorescence

Cells (6 × 10^5^/500 μl medium) were grown for 24 h in a Lab-Tek II chamber slide with an eight-well glass slide (Thermo Fisher Scientific, Rockford, IL, USA). After the addition of drugs for 24 h, cells were washed twice with PBS and fixed in 3.7% formaldehyde for 20 min. They were then washed three times with PBS for 5 min each. Cells were permeabilized with 0.25% Triton-X100 for 10 min, rewashed three times with PBS, and blocked for 30 min with 1% BSA and 0.02% NaN_3_ in PBS (blocking buffer). Rabbit anti-GRP78 primary antibody (polyclonal, Thermo Fisher Scientific) was added for 3 h at room temperature, and cells were washed three times for 10 min each with blocking buffer. Secondary antibody (donkey anti-rabbit IgG, PE-conjugated, Jackson ImmunoResearch Laboratories, West Grove, PA, USA) was added to the cells for 1 h, and following three washes of 5 min each with blocking buffer, primary anti-CREB3L1 antibody (mouse monoclonal IgG1; Santa Cruz Biotechnologies, Dallas, TX, USA) was added overnight at 4°C. After three washes with blocking buffer, cells were incubated with the secondary anti-mouse Alexa 488 antibody (donkey anti mouse IgG; Invitrogen, Waltham, MA, USA) for 1 h at room temperature. Cells were washed three times with PBS for 20 min and once with water and 300 μl DAPI solution, and NucBlue Fixed cell stain (Molecular Probes Invitrogen) was added to each well for nuclear counterstaining. Samples were rinsed once with PBS and mounted with Prolong Gold anti-fade reagent (Molecular Probes). Antibody binding was visualized under a confocal microscope at 63 × magnification (ApoTome.2, Carl Zeiss, Jena, Germany). Data were analyzed using ZEN2 Pro software (Zen, Jena, Germany). ImageJ software was used to calculate fluorescence intensity for CREB3L1 and GRP78 expression in the breast cancer cell lines in five different slides for each, before and after the addition of doxorubicin and paclitaxel.

### Animal Studies

All mouse experiments were performed in accordance with protocols approved by the Institutional Animal Care and Use Committee of Tel Aviv University (Tel Aviv, Israel, no. 01-18-056). The mouse metastasis model was adapted from Iorns et al. (31). Female Balb-C nude mice (Envigo, Rehovot, Israel), 4–6 weeks old, were used for the metastasis studies, in six groups of 5 mice each, as follows: (1) MDAMB231 treated i.p. with saline (control); (2) MDAMB231 treated with doxorubicin; (3) MDAMB231 treated with paclitaxel; (4) MDAMB231 CREB3L1-KO treated with saline (control); (5) MDAMB231 CREB3L1-KO treated with doxorubicin; (6) MDAMB231 CREB3L1-KO treated with paclitaxel. Mice were intravenously injected with either MDAMB231 cells (3.5 × 10^6^/200 μl PBS) or CREB3L1-KO MDAMB231 cells (3 × 10^6^/200 μl PBS) and inoculated peritoneally with doxorubicin (5 mg/kg) and paclitaxel (25 mg/kg) once a week. In a preliminary study, we determined the number of MDAMB231 and CREB3L1 KO cells injected to mice to achieve larger metastasis. Metastases in lungs were evaluated histologically 21 days after cell injection.

### Histological Determination

Eighteen lung tissue samples were serially cut into 5 μm sections with a cryotome. For immunohistochemistry (IHC) examination, sections were routinely stained using hematoxylin and eosin (H&E, 10 ×). The presence of tumors, size and number of foci in the lungs were measured by an expert pathologist at Rabin Medical Center in three mice lungs samples per group (six groups). To determine CREB3L1 and GRP78 expression in the metastatic mouse lung lesions, IHC slides were stained with anti-CREB3L1 and anti-GRP78 antibodies. In brief, paraffin-embedded sections were treated with xylene and washed sequentially with 95, 70, 50, and 30% ethanol, followed by a pure water wash. Endogenous peroxidase activity was blocked by incubating slides with 3% hydrogen peroxide for 5 min. For antigen retrieval, slides were washed with PBS and incubated with 50 mM Tris/20 mM EDTA, pH 9, in a microwave for 7 min. Sections were blocked with blocking buffer for 1 h. Anti-GRP78 and anti-CREB3L1 antibodies, followed by DAPI and mounting medium, were used as described for immunofluorescence staining. IHC results were captured as fluorescent field images (40 ×) using a confocal microscope and analyzed using ZEN2 Pro software.

### Statistical Analysis

All experiments were repeated at least three times. Data are expressed as mean ± SD. Student *t*-test was used to compare two groups. ANOVA followed by Tukey–Kramer multiple comparison method for comparisons involving more than two experimental groups. A *p* < 0.05 was considered statistically significant.

## Results

### Chemotherapy Induces Cell Surface GRP78 and CREB3L1 Expression

The expression of cell surface GRP78 and its downstream transcription factor, CREB3L1, was induced by the addition of doxorubicin and paclitaxel to cultures of ER positive MCF7 (*n* = 11), HER2-neu positive BT474 (*n* = 4), and triple-negative MDAMB231 (*n* = 8) and MDAMB468 (*n* = 7) breast cancer cell lines. At 24 h after addition of the drugs, the percentage of cells expressing both proteins was evaluated by FACS.

**Figure 1:**
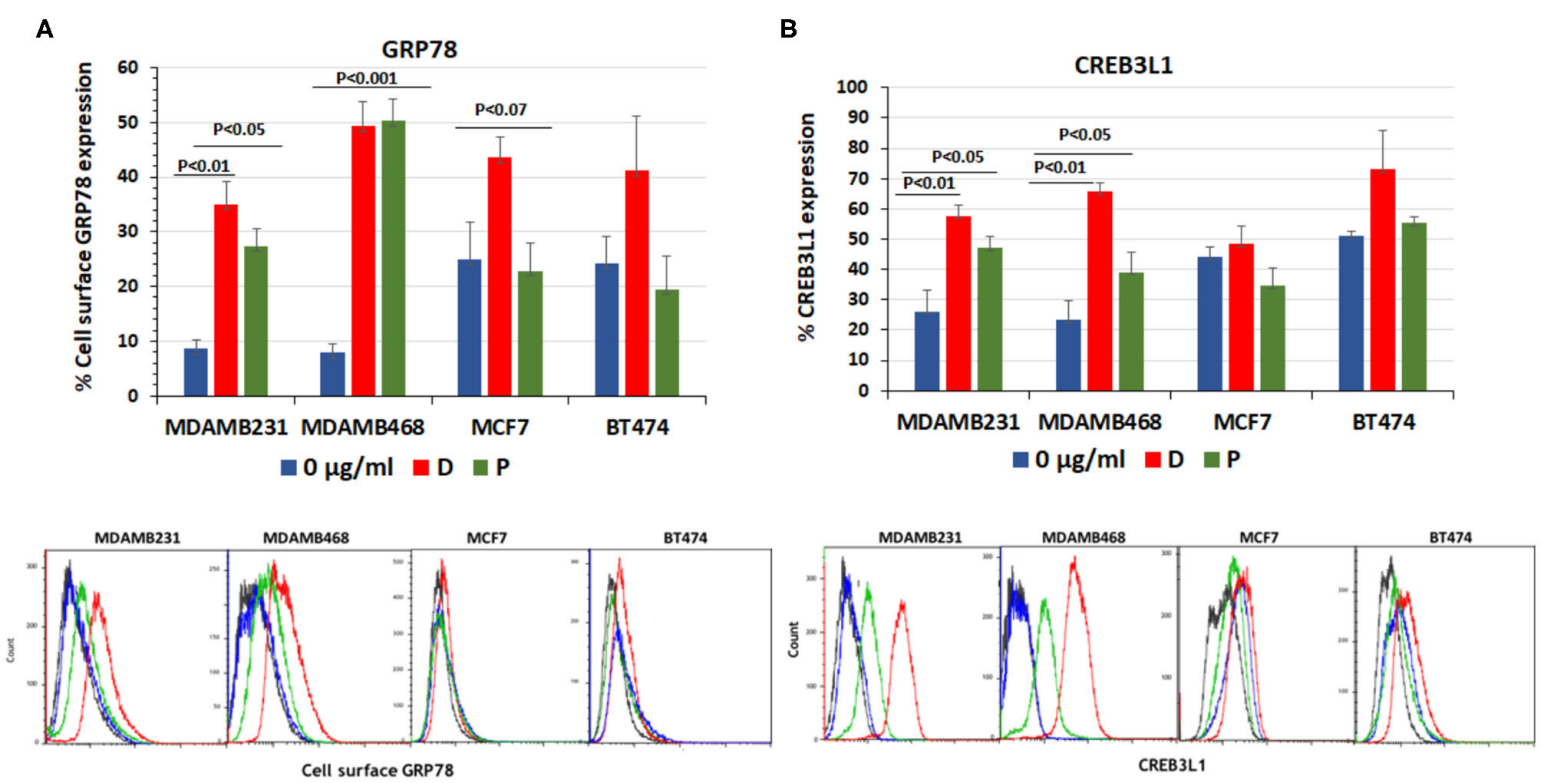
Doxorubicin and paclitaxel increased cell surface GRP78 and CREB3L1 expression in TNBC cells. FACS analysis of cell surface following addition of doxorubicin (D) (0.01 μg/ml) and paclitaxel (P) (0.01 μg/ml) to MDAMB231, MDAMB468, MCF7, and BT474 cell lines: **(A)** GRP78, **(B)** CREB3L1. Representative FACS histograms are presented below the respective graphs. In blue- Control, in red-doxorubicin and in green- paclitaxel.

The baseline percentage of cell-surface GRP78 and CREB3L1 expression was higher in MCF7 and BT474 cell lines than in TNBC cell lines. Following the addition of chemotherapy (31), GRP78 values showed no significant increase from baseline in MCF7 cells (24.8 ± 7.0% to 44.2 ± 4%, *p* < 0.08) and BT474 cells (24.3 ± 5% to 41.03 ± 10, *p* < 0.2) (Figure 1A). In contrast, in the triple negative MDAMB231 line, GRP78 expression significantly increased from 8.6 ± 1.7% at baseline to 35.1 ± 4.1% after the addition of doxorubicin (*p* < 0.01) and to 27.4 ± 3.1% after the addition of paclitaxel (*p* < 0.05). In the MDAMB468 cell line, baseline GRP78 increased from 7.9 ± 1.5% at baseline to 49.27 ± 4.6% after the addition of doxorubicin and to 50.3 ± 3.9% after the addition of paclitaxel (*p* < 0.001 for both, Figure 1A). Similarly, CREB3L1 expression was not affected significantly by doxorubicin or paclitaxel in either the MCF7 or BT474 cell lines (Figure 1B). The non-significant protein increase in MCF7 and BT474 is related to the variations in the results obtained from different samples and due to the higher expression of both proteins at the baseline.

### Chemotherapy Prevents Migration of TNBC Cells

**Figure 2:**
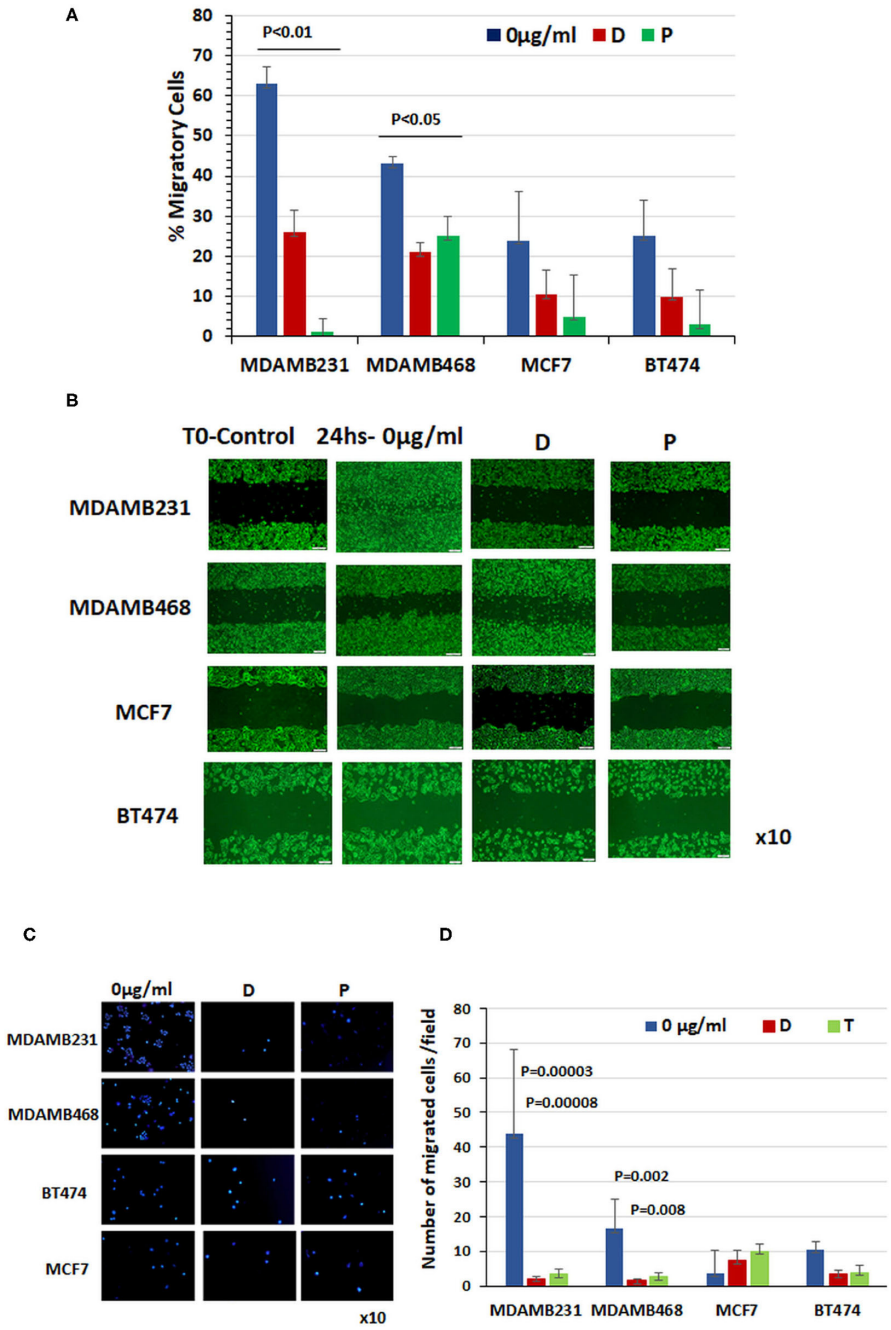
Chemotherapy prevented migration of triple-negative breast cancer cells. **(A)** Percentage of cell migration determined with monolayer gap closure assays. **(B)** Representative snapshots taken under a light microscope immediately after wound generation (10 ×, scale bar = 200 μm) and 24 h after addition of chemotherapy drugs to cultures. **(C)** Representative photographs of DAPI-stained cells obtained under a light microscope (10 ×). Note the cell migration through a transwell after 24 h treatment with doxorubicin or paclitaxel compared to untreated control cells. **(D)** Quantitation of migrated cells in each cell line at baseline and 24 h after administration of doxorubicin and paclitaxel (results of three different experiments ± SE).

Having demonstrated that chemotherapy significantly induces cell surface GRP78 and CREB3L1 expression specifically in TNBC cells, we evaluated the migratory capacity of these cells relative to ER positive and HER2-neu positive cell lines (Figure 2).

Before treatment, MCF7 and BT474 cells expressing cell-surface GRP78 and CREB3L1 showed lower migratory capability than TNBC cells expressing significantly lower levels of the proteins. Specifically, the baseline percentage of migrating cells was 24 ± 12% for MCF7 (*n* = 11) and 25 ± 9% for BT474 (*n* = 9) compared to 63 ± 8.2% for MDAMB231 (*n* = 10) and 43 ± 2% and for MDAMB468 (*n* = 13) (Figure 2A). The addition of chemotherapy drugs had little effect on the migratory capability of MCF7 cells (3.6 ± 2.2%) and BT474 cells (10.5 ± 6.77%), but it prevented migration of TNBC cells. The percentage of cell migration after doxorubicin treatment decreased to 26 ± 5.6% in MDAMB231 cells and to 21 ± 2.3% in MDAMB468 cells, and after paclitaxel, to 1 ± 3.4% and to 25 ± 5%, respectively (Figure 2A). Data from a representative monolayer gap closure assay showing differences in the migratory activities of the various cell lines are presented in Figure 2B. Representative photographs of DAPI-stained cells that migrated through a transwell after 24 h treatment with doxorubicin or paclitaxel are shown in Figure 2C compared to untreated control cells. These findings corroborate the scratch migration assay showing a significant difference in the number of migrating cells at baseline between the triple-negative MDAM231 cell line (43.7 ± 24.2) and both the ER positive cell line (3.6 ± 2.2; *p* = 0.05) and HER2-neu positive cell line (10.5 ± 6.77; *p* = 0.0002), with a significant reduction following treatment only in the triple-negative cells: by 20-fold after doxorubicin (*p* = 0.00003) and 13-fold after paclitaxel (p = 0.00008) in MDAMB231 cells, and by 10-fold (*p* = 0.002) and 6-fold (*p* = 0.009), respectively, in MDAMB468 cells (Figure 2D). BT474 and MCF7 cells showed a similar pattern of migration at baseline, with non-significant changes after treatment (Figure 2D).

### Chemotherapy Induces Nuclear Translocation of CREB3L1 in TNBC Cells

**Figure 3:**
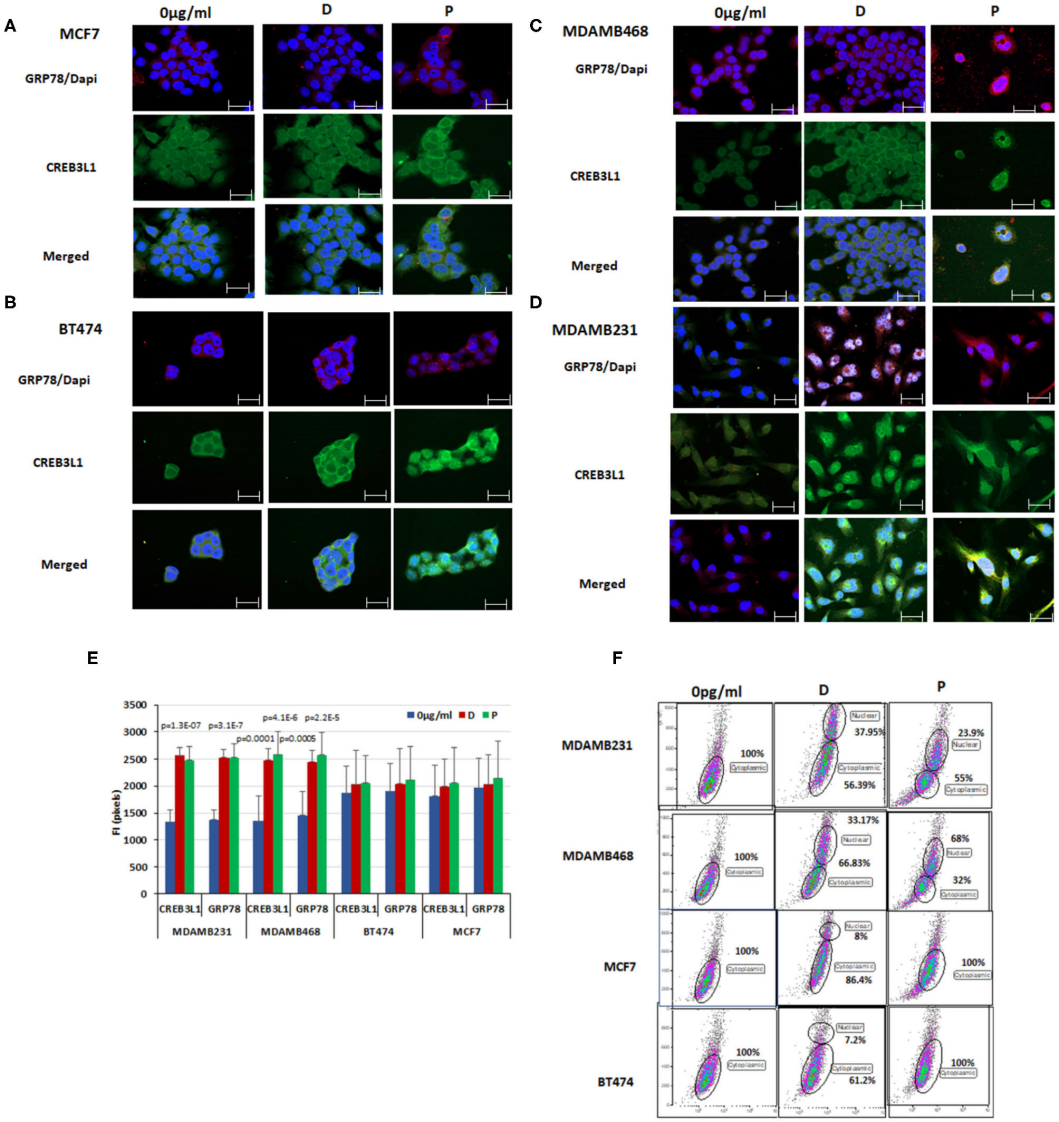
Chemotherapy induced nuclear translocation of CREB3L1 in MDAMB231 cells. Immunofluorescence analysis of GRP78 and CREB3L1 expression 24 h after cell treatment with doxorubicin and paclitaxel: **(A)** MCF7 cells, **(B)** BT474 cells, **(C)** MDAMB468 cells, **(D)** MDAMB231 cells. Note GRP78 staining (red), CREB3L1 staining (green), and merged staining (yellow) by cell type (63 × magnification, scale bar = 10 μm). **(E)** Fluorescence intensity (FI) of CREB3L1 and GRP78 expression. **(F)** Dot Blot FACS analysis of the cytoplasmic and nuclear expression of CREB3L1 in the different breast cancer cell lines.

Immunofluorescence and FACS analysis was used to evaluate the expression patterns of GRP78 and CREB3L1 in the four breast cancer cell lines. GRP78 and CREB3L1 expression and fluorescence intensity before and after chemotherapy are presented in Figure 3.

GRP78 (red staining) and CREB3L1 (green staining) baseline levels were higher in MCF7 and BT474 cells (Figures 3A,B) than in MDAMB231 and MDAMB468 cells (Figures 3C,D). Treatment with doxorubicin or paclitaxel promoted GRP78 and CREB3L1 expression in all cells. CREB3L1 was observed principally in the cytoplasm regardless of whether or not chemotherapy was administered. The triple negative cell lines showed a specific pattern of protein expression following doxorubicin or paclitaxel treatment characterized by the presence of CREB3L1 in the nucleus, indicating its translocation from the cytoplasm (Figure 3D). Administration of chemotherapy induced a significant increase in expression of both proteins in the same cells with a similar pattern: yellow staining as a result of merging of GRP78 (red) and CREB3L1 (green). In contrast to TNBC cells, FACS analysis of MCF7 and BT474 cells yielded both proteins but not in the nucleus. A small portion of cells expressing nuclear CREB3L1 was observed after doxorubicin treatment but not after paclitaxel.

The results obtained from different samples were corroborated by fluorescence intensity measurements using ImageJ software. Following treatment, the fluorescence intensity of CREB3LI increased significantly (>18-fold) in MDAMB231 cells (*p* = 1.3 × 10^−7^ for doxorubicin, *p* = 6.8 × 10^−6^ for paclitaxel) and MDAMB468 cells (*p* = 0.0001 for doxorubicin, *p* = 4.1 × 10^−6^ for paclitaxel). Similar effects were noted for GRP78 in both MDAMB231 cells (*p* = 3.1 × 10^−7^ for doxorubicin, *p* = 4.1 × 10^−6^ for paclitaxel) and MDAMB468 cells (*p* = 0.0005 and *p* = 2.2 × 10^−5^, respectively). In BT474 and MCF7 cells, there was no change in the fluorescence intensity of CREB3L1 or GRP78 from before to after treatment with either chemotherapeutic agent (Figure 3E).

FACS analysis was used to detect nuclear CREB3L1, using a previously published protocol (32) (Figure 3F). CREB3L1 at baseline was expressed only in the cytoplasm in all four cell lines. After treatment, nuclear CREB3L1 was detected in the TNBC cell lines only. In MDAMB231 cell line, the number of cells expressing CREB3LI in the nucleus increased by 37.95 ± 1.05% after the addition of doxorubicin and by 50.29 ± 0.66% after the addition of paclitaxel. The respective increase for each drug in MDAMB468 cell line was 33.17 ± 1.85% and 32 ± 3.64%. In MCF7 and BT474 cell lines, CREB3L1-expressing cells were found only in the cytoplasm after paclitaxel treatment. A small albeit significant (*p* < 0.03) percentage of cells expressed nuclear CREB3L1 after doxorubicin treatment (increase of 13.09 ± 1.75% in MCF7 cells, 12.24 ± 3.11% in BT474 cells).

### Chemotherapy Drugs Regulate CREB3L1 and Cell-Surface GRP78 Expression

**Figure 4:**
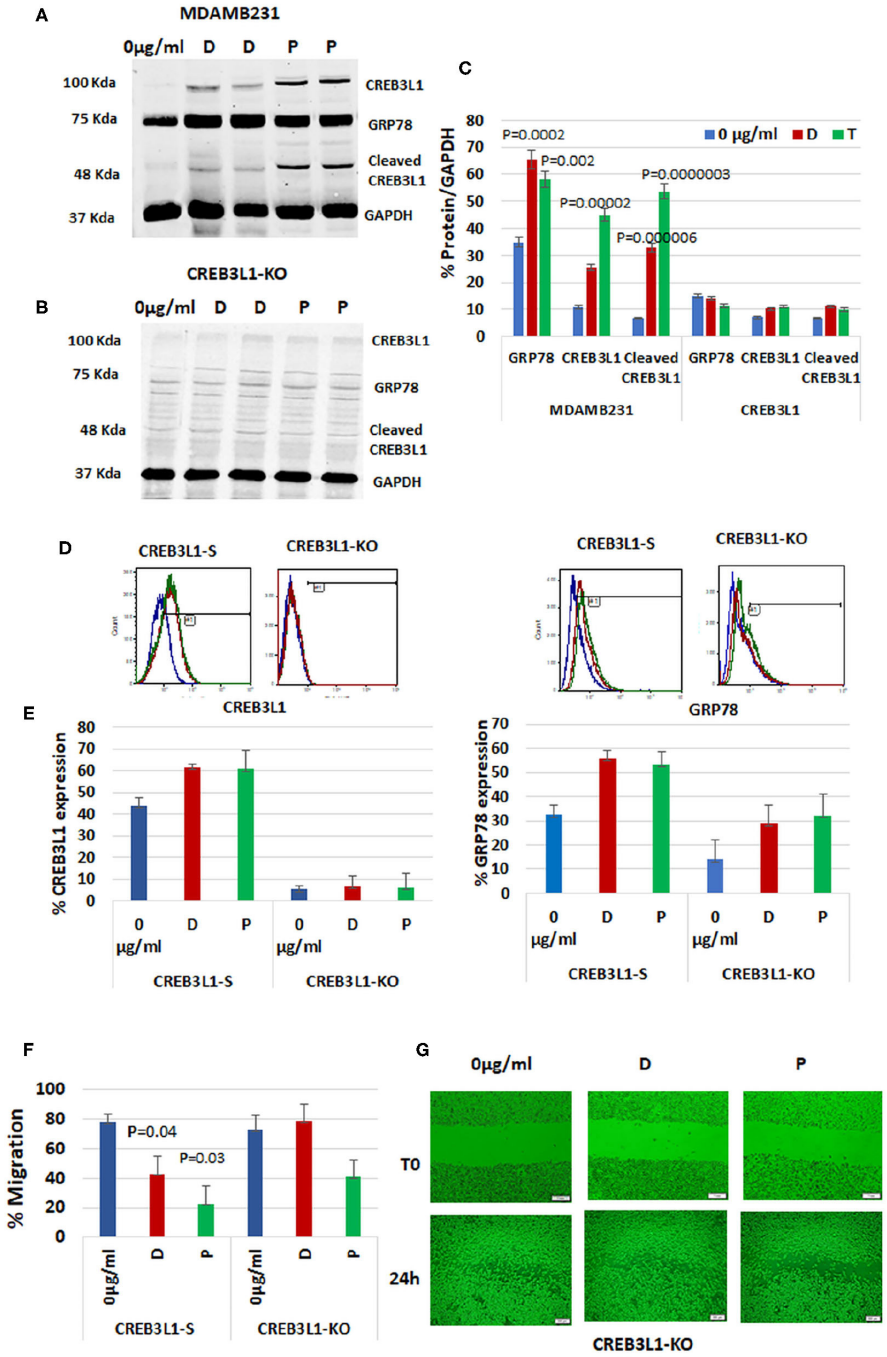
CREB3L1-KO in aggressive TNBC cells revokes prevention of migration. CREB3L1 was depleted using CRISPR/Cas9 in MDAMB231 cells. **(A)** Representative blot showing a significant increase in GRP78, CREB3L1, and cleaved CREB3L1 expression in doxorubicin (D) or paclitaxel (P) treated MDAMB231. **(B)** Representative blot showing lack of GRP78 and CREB3L1 expression in CREB3L1-KO cells. **(C)** Percentage of GRP78, CREB3L1, and cleaved CREB3L1 calculated vs. GAPDH (*n* = 3). **(D)** Representative FACS histograms showing cell-surface GRP78 and CREB3L1 expression in scrambled gRNA (CREB3L1-S) control-transfected and CREB3L1-KO-transfected cells. **(E)** Percentage of cell surface GRP78 and CREB3L1 expression in CREB3L1-S and CREB3L1-KO cells (*n* = 5). **(F)** Percentage of cell migration of CREB3L1-S and CREB3L1-KO cells treated with doxorubicin (D) or paclitaxel (P). **(G)** Representative snapshots taken under a light microscope (10 ×, scale bar = 200 μm) following wound generation in CREB3L1-KO cells and 24 h after the addition of chemotherapy drugs corroborating CREB3L1 anti-metastatic activity.

To demonstrate the impact of CREB3L1, the downstream transcription factor of GRP78, on cell migration, we depleted it from the most aggressive MDAMB231 TNBC cells using CRISPR/Cas9 technology. First, wild-type MDAMB231 cells were compared with CREB3L1-KO cells for CREB3L1 and GRP78 expression (Figure 4). The MDAMB231 blots showed a significant increase in expression of CREB3L1 (~100 kDa) and GRP78 (78Kda) after doxorubicin treatment (by +2.3-fold) and after paclitaxel treatment (by +4.1-fold) (*p* = 0.00002). Total GRP78 expression increased by 1.9-fold with doxorubicin (*p* = 0.0002) and by 1.7-fold with paclitaxel (*p* = 0.002). At ~55Kda, we observed the cleaved CREB3L1, which was increased significantly, by 5-fold with doxorubicin (*p* = 0.000006) and by 8-fold with paclitaxel (*p* = 0.0000003) (Figure 4A). In the CREB3L1-KO cells, the expression of CREB3L1, and consequently of GRP78, was minimal or lacking in all conditions, as expected (Figure 4B). The quantitation of protein expression from three different experiments is shown in Figure 4C.

FACS analysis was used to differentiate cell-surface GRP78 from total GRP78 in the western blot (Figure 4D). MDAMB231 cells transfected with a CRISPR/Cas9 scrambled vector (CREB3L1-S) exhibited an increase in cell-surface GRP78 and CREB3L1 after drug treatment. However, the change was not significant owing to the stress induced by the transfection itself, as reflected by the increased cell-surface GRP78 and CREB3L1 at baseline (Figure 4E). In CREB3L1-KO cells, very low or null expression of CREB3L1 and GRP78 was detected before and after chemotherapy (Figure 4E).

Immunofluorescence of CREB3L1-KO cells revealed null expression of CREB3L1 and GRP78, confirming the FACS and western blot results (Supplementary Figure 1).

### CREB3L1-KO in Aggressive TNBC Cells Revokes Suppression of Migration

Migration patterns of cells transfected with scrambled CREB3L1 or CREB3L1-KO vector before and after doxorubicin or paclitaxel treatment are depicted in Figure 4F. In CREB3L1-S cells, there was a significant decrease in migration after 24-h treatment with doxorubicin (*p* = 0.04) or paclitaxel (*p* = 0.03). In contrast, CREB3L1-KO cells failed to respond to the drugs and showed increased migration even after treatment (Figure 4G; representative images obtained under a light microscope).

To determine if CREB3L1 affects cell proliferative capacity, we performed colony formation assays of MDAMB231 and CREB3L1-KO cells. The results of three experiments showed no differences at baseline in the number of colonies between MDAMB231 wild-type cells and CREB3L1-KO cells. Following treatment with doxorubicin or paclitaxel, the number of colonies was significantly reduced (Supplementary Figure 2).

### Chemotherapy Fails to Prevent Metastasis in Mice Injected With MDAMB231 CREB3L1-KO Cells

**Figure 5:**
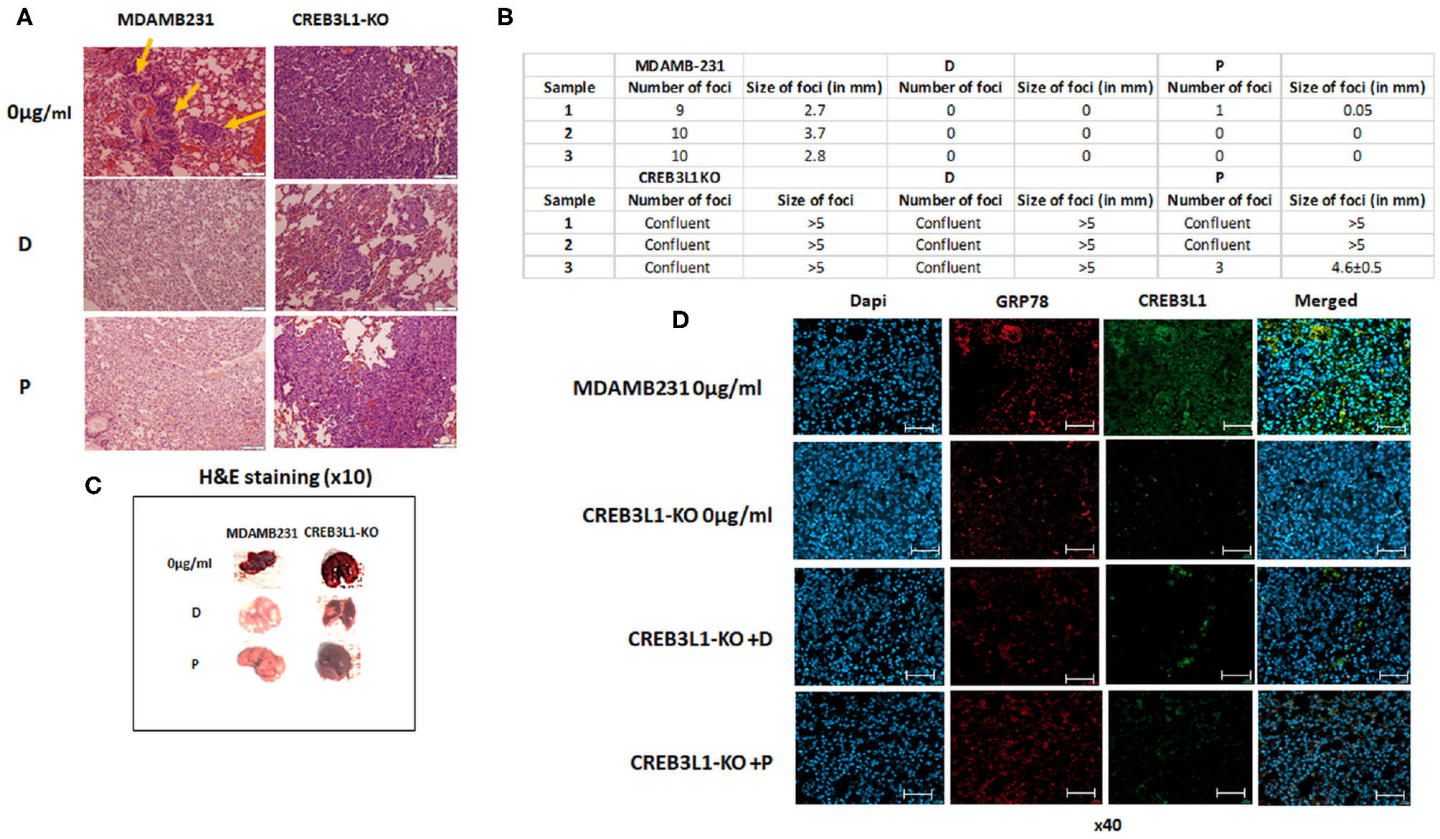
Chemotherapy fails to prevent metastasis in mice injected with MDAMB231 CREB3L1-KO cells. **(A)** Representative histological lung sections stained with H&E showing metastasis in mice injected IV with MDAMB231 control cells, compared to the group inoculated with CREB3L1-KO cells and after chemotherapy. **(B)** Number and size (in mm) of lung foci were assessed manually in three mice in each group **(C)** Lungs extracted from mice. **(D)** Histological sections showing metastasis in lungs of mice inoculated with MDAMB231 or CREB3L1-KO cells. Immune staining for CREB3L1 (green) and GRP78 (red). Nuclei of cells stained with DAPI. Immunofluorescence images obtained under a confocal microscope and analyzed with Zen2Pro Zen. Inc software (×40, scale bar = 200 μm).

An *in vivo* mouse model was employed to investigate the involvement of CREB3L1 in the metastatic potential of cells. Intravenous injection of MDAMB231 wild-type or MDAMB231-KO cells induced lung metastases 21 days after inoculation. H&E staining of histological sections of lungs from MDAMB231-injected mice revealed several metastatic foci throughout. Viable adenocarcinoma cells were identified in all foci. Mice inoculated with MDAMB231-KO cells showed massive metastatic involvement of parenchymal and pleural tissue in whole lung (Figure 5A).

Following doxorubicin and paclitaxel treatment of mice inoculated with wild-type MDAMB231 cells, lung samples extracted from different animals showed no evidence of metastatic disease. In contrast, mice injected with MDAMB231/CREB3L1-KO and given chemotherapy showed multifocal parenchymal metastases and large metastatic tumors with increased pleomorphism and intravascular invasion (Figure 5A). Consistent with the microscopic observations, treatment with doxorubicin or paclitaxel reduced almost completely the number and size of the metastatic foci in lungs of mice transfused with wild type MDAMB231. In contrast, CREB3L1 gene ablation canceled drugs effect (Figure 5B). Further macro examination of the lungs of MDAMB231 control and CREB3L1-KO mice before and after chemotherapy (Figure 5C) revealed that the lungs of the CREB3L1-KO mice were significantly bigger and more metastatic. After treatment, the lungs of the control mice returned to their normal size and color whereas the lungs of the CREB3L1-KO-injected mice showed minimal change.

Histological lung sections were stained for GRP78 and CREB3L1 before and after treatment. Sections from control mice before treatment disclosed low expression of both proteins (Figure 5D). Since there was no evidence of disease after treatment, no staining was performed. In lungs of CREB3L1-KO-injected mice, metastatic foci displayed lack of CREB3L1 and GRP78 expression both before and after treatment (Figure 5D).

## Discussion

CREB3L1, a member of the CREB/ATF family of transcription factors, is the most recently identified member of the UPR pathway. In its mature activated form, it functions as a transcription factor to promote the expression of GRP78 (33, 34). In the present study, we investigated the impact of chemotherapy on CREB3L1 and cell-surface GRP78 expression, and its association with the development of breast cancer metastasis.

Initially, TNBC cell lines (MDAMB468 and MDAMB231) were compared with ER positive and HER2-neu positive cell lines (MCF7 and BT474) for CREB3L1 and cell-surface GRP78 expression before (baseline) and after chemotherapy. The TNBC cell lines displayed relatively lower baseline levels of CREB3L1 than the others, consistent with previous findings of frequently upregulated CREB3L1 in ER positive and HER2-neu positive breast cancers, but not in TNBC (29). Studies have reported DNA hypermethylation in several key CpG cites within the *CREB3L1* gene in TNBC cells which appears to be strongly correlated with reduced expression of the protein (29, 35, 36). Additionally, low CREB3L1 expression in TNBC cells is significantly associated with more aggressive high-grade tumors, potentially contributing to a highly metastatic phenotype (28, 29). Accordingly, the MDAMB231 cell line, which expresses lower levels of CREB3L1 at baseline, has more aggressive and metastatic features than other cell lines (29). The MDAMB468 cells which also express lower levels of CREB3L1 at baseline are highly proliferative with high Ki67 (37). In our experiments, the administration of chemotherapy (doxorubicin/paclitaxel) induced a significant increase in CREB3L1 protein expression in TNBC cell lines but not in ER positive or HER2-neu positive cell lines.

To confirm that the activation of CREB3L1 was mediated through the UPR pathway, we examined the correlation between CREB3L1 expression in each subtype with the expression of the key UPR molecule, GRP78, focusing on cell-surface GRP78 (38). The results demonstrated a significant increase in the expression of both cell-surface GRP78 and CREB3L1 in response to doxorubicin and paclitaxel specifically in the TNBC cell lines. The findings support the hypothesis that these chemotherapeutic agents trigger the UPR mechanism through effects on CREB3L1.

Cell migration is one of the hallmarks of tumor metastatic potential. Therefore, after demonstrating the impact of chemotherapy on the expression of GRP78 and CREB3L1 in TNBC, we examined the correlations between the protein expression patterns and the tumor migration capacities using two different techniques. Our experiments revealed that doxorubicin and paclitaxel inhibited migration specifically in TNBC cell lines, which responded to chemotherapy with increased levels of CREB3L1 and cell-surface GRP78. Conversely, in the other cell lines in which post-therapy CREB3L1 or cell-surface GRP78 levels did not change, we observed only minor alterations in migration or invasion.

In the TNBC cell lines, we observed differences in response to drugs in cell migration function. Cell migration was almost completely inhibited by paclitaxel in the highly metastatic MDAMB231 while doxorubicin inhibited moderately cell migration. MDAMB468 migration was inhibited moderately with both drugs, compared to MDAMB231. We assume that the drug sensitivity variations between both cell lines stem from the differences in the drugs mechanisms of action. Paclitaxel is identified as an inhibitor of cell migration at concentrations used in this study by significantly suppressing microtubule dynamics (39, 40). Doxorubicin intercalates into the DNA and blocks the topoisomerase-II-mediated DNA repair leading to proliferation blockade (40).

CREB3L1 is an endoplasmic reticulum transmembrane protein that is activated via site-1 protease (S1P) and site-2 protease (S2P) cleavage in the Golgi apparatus and subsequently translocates to the nucleus (28, 41). Immunofluorescence and FACS evaluation revealed GRP78 and CREB3L1 protein expression in the same tumor cells in the doxorubicin/paclitaxel treated TNBC cell only lines. In addition, nuclear translocation of CREB3L1 after chemotherapy was observed only in the TNBC cells.

Western blot analysis of MDAMB231 cells after doxorubicin or paclitaxel treatment disclosed a cleaved (activated) form of CREB3L1, apparently induced by the chemotherapy according to our collective results. Activated CREB3L1 functions as a transcription factor, affecting responsive elements of the endoplasmic reticulum and cyclic AMP to increase GRP78 expression (24), thereby stimulating GRP78 translocation to the cell surface (21, 38, 42). In the present study, only MDAMB231 and MDAMB468 cells, which showed a higher expression of cell-surface GRP78 and nuclear CREB3L1 in response to chemotherapy, exhibited a significantly reduced migratory profile. Ward et al. (29) found that in high-grade breast tumors, CREB3L1 was localized mainly in the cytoplasm, but in low-grade breast tumors, it translocated to the nucleus. In our experiments, CREB3L1 translocation to the nucleus was correlated with a switch of the TNBC cells to a less migratory/invasive phenotype.

To further confirm the involvement of cell-surface GRP78 and its transcription factor CREB3L1 in the migratory function of cancer cells, we depleted CREB3L1, using CRISPR/Cas9 technology, in the MDAMB231 cell line (43, 44). Whereas, CREB3L1 wild-type cells had responded to chemotherapy, the CREB3L1-KO cells demonstrated resistance to doxorubicin and paclitaxel and strong cell migratory capacity. Together, these results reinforced the important inhibitory role of CREB3L1 in cell migration, coinciding with earlier studies showing that re-expression of CREB3L1 in CREB3L1-silenced cells reduced *in vitro* cell migration and invasion under hypoxic conditions (29). In our study, CREB3L1 expression corresponded to cell-surface GRP78 expression in these cells, indicating that CREB3L1 responded to chemotherapy through the UPR pathway.

Unlike the impact of CREB3L1 on migration, in colony formation experiments, CREB3L1 expression and activation had no effect on the proliferative capacity of the cells. In our study, in the MDAM231 cell line, chemotherapy significantly reduced colony formation regardless of CREB3L1 status. A study by Naviglio et al. (45) showed that Interferon alpha induced moderated apoptosis in epidermoid cancer cells through downregulation in CREB phosphorylation/activation without changes in its total expression. In contrast, previous reports indicated that doxorubicin blocks the proliferation of cancer cells through the proteolytic activation of CREB3L1 (28).

Our *in vivo* results using the CREB3L1-KO TNBC cell line confirmed the results obtained *in vitro*, supporting the proposed concept that CREB3L1 is required to prevent metastatic spread. The lungs of the mice that had been injected intravenously with the triple-negative MDAMB231 CREB3L1-KO cells showed massive parenchymal and pleural involvement occupying the whole lung tissue. Interestingly, the lungs of mice inoculated with wild-type MDAMB231 with low CREB3L1 expression displayed several foci with significantly fewer metastatic lesions than the lungs of mice injected with the CREB3L1-KO cells. Moreover, mice injected with wild-type MDAMB231 responded to treatment with either doxorubicin or paclitaxel, resulting in complete disappearance of the metastases. Mice injected with CREB3L1-KO cells did not respond to treatment. Immunohistology sections confirmed the absence of CREB3L1 and GRP78 both before and after chemotherapy administration. Our results are consistent with a previous study in a rat model showing that CREB3L1-expressing cells failed to form metastases, and CREB3L1 re-expression in CREB3L1-silenced cells initially led to the formation of large tumors followed by 70% metastasis regression (22).

One report described CREB3L1 as a promoter of the metastatic process in tumors that have activated both protein kinase RNA-like endoplasmic reticulum kinase (PERK) signaling and epithelial-to-mesenchymal programming (46). However, our data demonstrated that UPR activation by chemotherapy through induction of CREB3L1 and cell surface GRP78 prevented the metastatic process, both *in vitro* and *in vivo*.

The present results regarding cell-surface GRP78 expression contradict some previous studies and support others (14, 47). The contradictory results may be attributed at least partly to the protective role of mild UPR activation in breast cancer which contributes to an aggressive cell profile (20, 21). In contrast, hyperactivation of the UPR pathway in cancer cells treated with GRP78-inducing chemotherapy agents may trigger a conversion from a cytoprotective to a significantly less metastatic-aggressive profile (48, 49). In addition, we observed a different impact of UPR activation, confirmed by CREB3L1 and cell-surface GRP78 expression, in the different breast cancer subtypes. The different behaviors have a great impact on the type of systemic therapy selected.

This study demonstrated that chemotherapy activated CREB3L1 and enhanced cell-surface GRP78 expression, resulting in a reduced metastatic potential of TNBC cells. Previous studies of pancreatic cancer found GRP78 to be important in promoting tumor-initiating cell populations (tumor stem cells) (19). Doxorubicin and paclitaxel administered to patients with TNBC disease impaired the migration of differentiating cells, but it is possible that a clone of tumor stem cells expressing cell-surface GRP78 was not harmed (19). In a previous study of ours, we found that residual breast tumor cells after neoadjuvant therapy harbor positive cell-surface GRP78 (50). The residual tumor with high surface GRP78 expression may represent a resistant clone with stemness features that do not respond to chemotherapy (19).

The current study elucidates a potential new strategy for the treatment of metastatic TNBC based on the modulation of CREB3L1 and cell-surface GRP78 expression by chemotherapy in addition to GRP78-targeted drugs for the elimination of tumor-initiating clones.

## Data Availability Statement

The datasets generated for this study are available on request to the corresponding author.

## Ethics Statement

The animal study was reviewed and approved by Institutional Animal Care and Use Committee of Tel Aviv University (Tel Aviv, Israel, no. 01-18-056).

## Author Contributions

AR and RY conceived the experiments and wrote the paper. JL and SM carried out the *in vitro* and *in vivo* experiments. LH analyzed the *in vivo* data. AR analyzed the data. TB-Z supervised the CRISPR technology. All authors contributed to the article and approved the submitted version.

## Conflict of Interest

The authors declare that the research was conducted in the absence of any commercial or financial relationships that could be construed as a potential conflict of interest.

## Abbreviations

CREB3L1: cyclic AMP responsive element binding protein 3-like 1
FACS: fluorescence activated cell sorting
GFP: green fluorescent protein
GRP78: Glucose-regulated protein 78
KO: knock-out
TNBC: triple-negative breast cancer
UPR: unfolded protein response.
