# Supplementary material for: Chemotherapy Controls Metastasis Through Stimulatory Effects on GRP78 and Its Transcription Factor CREB3L1
Source: Front Oncol. 2020 Sep 11;10:1500. doi: 10.3389/fonc.2020.01500 (PMC7518037; doi:10.3389/fonc.2020.01500)
Supplement: Supplementary file 1 [file Table_1.docx]

**Chemotherapy controls metastasis through stimulatory effects on GRP78 and its transcription factor CREB3L1**

Annat Raiter^1,4,^**^*^**, Julia Lipovetski^1^, Lucila Hyman^2^, Shany Mugami^1^, Tali Ben-Zur^1,^, Rinat Yerushalmi^1,3,4,^**^*^**

^1^Felsenstein Medical Research Center, Petach Tikva, Israel; ^2^Department of Pathology, Rabin Medical Center, Beilinson Hospital, Petach Tikva, Israel; ^3^Davidoff Cancer Center, Rabin Medical Center, Petach Tikva, Israel; ^4^Sackler Faculty of Medicine, Tel Aviv University, Tel Aviv, Israel

**Supplementary material:**

**Figure 1**. **Immunofluorescence of CREB3L1-KO cells treated with doxorubicin (D) or paclitaxel (P).** Immunofluorescence of CREB3L1-KO cells demonstrated absence of CERB3L1 and GRP78 expression, before and after chemotherapy treatment. Cells (6×10^5^/500 μl medium) were grown for 24 hours in a Lab-Tek II chamber slide with an eight-well glass slide (Thermo Fisher Scientific, Rockford, IL, USA). At 24 hours addition of the drugs, cells were washed twice with PBS and fixed in 3.7% formaldehyde for 20 minutes. Next, cells were washed three times with PBS for 5 minutes each. Cells were permeabilized with 0.25% Triton-X100 for 10 minutes, rewashed three times with PBS, and subsequently blocked for 30 minutes with 1% bovine serum albumin (BSA) and 0.02% NaN_3_ in PBS (blocking buffer). After blocking, rabbit anti-GRP78 primary antibody (polyclonal, ThermoFisher Scientific) was added for 3 hours at room temperature. Cells were washed three times for 10 minutes each with blocking buffer, and secondary antibody (donkey anti-rabbit IgG, PE- conjugated, Jackson ImmunoResearch Laboratories, West Grove, PA, USA) was added for 1 hour. After 3 washes of 5 minutes each with blocking buffer, primary anti-CREB3L1 antibody (mouse monoclonal IgG1; Santa Cruz Biotechnologies, Dallas, TX, USA) was added overnight at 4°C. Cells were washed three times with blocking buffer and incubated with the secondary anti-mouse Alexa 488 antibody (donkey anti-mouse IgG; Invitrogen, Waltham, MA USA) for 1 hour at room temperature After washing, cells were stained with NucBlue Fixed cell stain (Molecular Probes Invitrogen) was added to each well for nuclear counterstaining. Samples were rinsed once with PBS and mounted with Prolong Gold anti-fade reagent (Molecular Probes). Antibody binding was visualized under a confocal microscope at 63× magnification (ApoTome.2, Carl Zeiss, Jena, Germany), and data analyzed using Zen2pro (Zen, Jena, Germany).


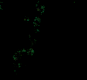


**CREB3L1-KO**

**0µg/ml D P**

**CREB3L1**

**GRP78**

**Dapi**

**Merged**


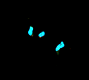

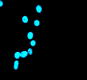

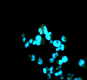

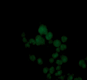

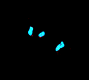

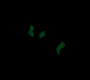

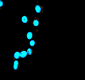

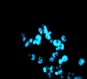


**x63**

**Figure 2:** **Colony formation of MDAMB231 and CREB3L1-KO cells treated with doxorubicin (D) or paclitaxel (P).** The effect of CREB3L1 expression on the tumor cell proliferative capacity was demonstrated by colony formation using MDAMB231 and MDAMB231 CREB3L1-KO cells. No differences in the proliferative capacity was observed at baseline and after chemotherapy between MDAMB231 and CREB3L1-KO cells. Doxorubicin and paclitaxel reduced significantly the number of colonies in both, wild type and KO cells (p<0.05). Tumor cells were seeded (1000 cells/well) in triplicate onto six-well plates for 24 hours in complete culture medium. Drugs were added as described above. Medium was replaced every other day, and after 2 weeks, colonies were stained using gentian violet. Photographs were taken and the results were calculated using Image-Pro Plus software (Media Cybernetics, Silver Spring, MD, USA). **(A)** Results of four experiments are presented as mean number of colonies ± SD. (B) Representative colony formation results in six-well plates.


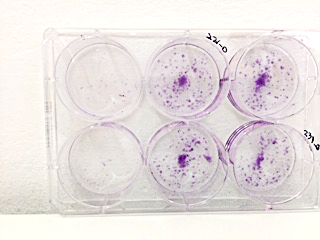

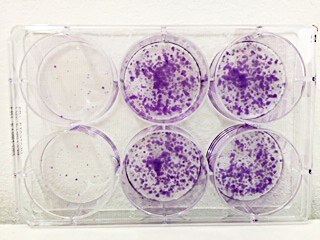

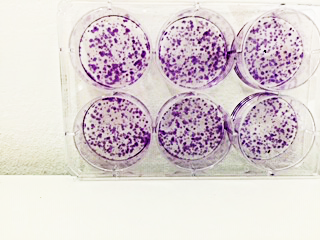


P<0.05

.
